# Protist predation can select for bacteria with lowered susceptibility to infection by lytic phages

**DOI:** 10.1186/s12862-015-0341-1

**Published:** 2015-05-07

**Authors:** Anni-Maria Örmälä-Odegrip, Ville Ojala, Teppo Hiltunen, Ji Zhang, Jaana KH Bamford, Jouni Laakso

**Affiliations:** Centre of Excellence in Biological Interactions, Department of Biological and Environmental, University of Jyväskylä, P.O. Box, 35, 40014 Jyväskylä, Finland; Department of Biosciences, University of Helsinki, P.O. Box 65, 00014 Helsinki, Finland; Department of Food and Environmental Sciences/Microbiology and Biotechnology, University of Helsinki, P.O. Box 65, 00014 Helsinki, Finland

**Keywords:** Antagonistic co-evolution, Bacteriophages, Host-parasite interaction, Multiple species interaction, Phage resistance, Phage-host interaction, *Pseudomonas fluorescens* SBW25, Protists, *Serratia marcescens*, Trade-off

## Abstract

**Background:**

Consumer-resource interactions constitute one of the most common types of interspecific antagonistic interaction. In natural communities, complex species interactions are likely to affect the outcomes of reciprocal co-evolution between consumers and their resource species. Individuals face multiple enemies simultaneously, and consequently they need to adapt to several different types of enemy pressures. In this study, we assessed how protist predation affects the susceptibility of bacterial populations to infection by viral parasites, and whether there is an associated cost of defence on the competitive ability of the bacteria. As a study system we used *Serratia marcescens* and its lytic bacteriophage, along with two bacteriovorous protists with distinct feeding modes: *Tetrahymena thermophila* (particle feeder) and *Acanthamoeba castellanii* (surface feeder). The results were further confirmed with another study system with *Pseudomonas* and *Tetrahymena thermophila.*

**Results:**

We found that selection by protist predators lowered the susceptibility to infections by lytic phages in *Serratia* and *Pseudomonas*. In *Serratia,* concurrent selection by phages and protists led to lowered susceptibility to phage infections and this effect was independent from whether the bacteria shared a co-evolutionary history with the phage population or not. Bacteria that had evolved with phages were overall more susceptible to phage infection (compared to bacteria with history with multiple enemies) but they were less vulnerable to the phages they had co-evolved with than ancestral phages. Selection by bacterial enemies was costly in general and was seen as a lowered fitness in absence of phages, measured as a biomass yield.

**Conclusions:**

Our results show the significance of multiple species interactions on pairwise consumer-resource interaction, and suggest potential overlap in defending against predatory and parasitic enemies in microbial consumer-resource communities. Ultimately, our results could have larger scale effects on eco-evolutionary community dynamics.

## Background

Consumer-resource interactions are important components of virtually all ecological communities and have been studied extensively as they may determine the stability and diversity of these communities [1]. Experimentally, these interactions are often studied with one-consumer one-resource systems. However, in complex natural communities, any given species interacts with multiple other species, and the potential interactions between species increase along with the number of species. Furthermore, in addition to ecological factors, evolution and eco-evolutionary feedbacks have been identified as important components of consumer resource dynamics [2]. In natural communities, bacteriovorous protists and lytic bacteriophages are the most prominent cause of bacterial death, each estimated to account for 50% of daily bacterial mortality [3,4]. Phages are typically highly host-specific while protist grazers choose their prey based on relatively non-specific features, such as prey size [5-7]. Another important difference between protists and phages is that a single protist grazer consumes multiple prey bacteria before reproducing, whereas a successful infection by a single parasitic phage results in multiple progeny from a single host bacterium. Defence mechanisms against these two types of enemies can be different: bacteria commonly acquire phage-resistance by altering the cell-surface receptors that the phage uses to gain entrance to the bacterial cell, and common ways to avoid protozoan predation include oversized morphology, cell clustering, biofilm formation, prevention of receptor-mediated phagocytosis, increase in bacterial motility, and secreting toxins against predators [8,9]. Due to these profound differences between phages and protists as consumers, they often pose differing selection pressures on bacterial traits. For example, exposure to the ciliate *Tetrahymena thermophila* has been shown to weaken the antagonistic arms-race co-evolutionary dynamics between *Pseudomonas fluorescens* and its associated parasitic bacteriophage [10]. However, besides this study, studies that address evolution against both phage and protozoan enemies simultaneously are all but nonexistent.

Pairwise antagonistic co-evolution is predicted to be shaped by the presence of additional interacting species, depending on how the traits that are selected for are correlated [11,12]. Indeed, there are plenty of studies showing negative correlations where the presence of one enemy reduces the evolutionary impact of another species [10,11,13-22]. Negative correlations resulting from multiple species interactions on pairwise antagonistic co-evolution are mostly due to trade-offs between defence mechanisms against multiple enemies, where a benefit from a change in one life-history trait is overridden by the disadvantage introduced by a change in another trait in a given environment [15,23]. One study investigating host-parasite and predator-prey interactions with bacteria, phages and protists was conducted with *Pseudomonas fluorescens*, its associated lytic bacteriophage and *Tetrahymena thermophila* [10]*.* The study showed that the presence of two bacterial enemies resulted in divergence of bacteria into specialized defenders against predators and parasites instead of a generalist defensive strategy against both enemies [10]. An example of positively correlated defence mechanisms against multiple enemies was shown in a study where *Pseudomonas syringae* was allowed to co-evolve with multiple phages: the bacterial hosts evolved resistance against multiple phages simultaneously, but this was accompanied by an associated cost on growth [24]. However, the enemies in this study were all bacteriophages rather than organisms from different taxonomic groups, and all had a parasitic relationship with the host.

In this study, we investigated how the presence of protist predators affects the susceptibility of bacterial to infection by lytic phage. To study this, we used two microbial systems, with either *Serratia marcescens* or *Pseudomonas fluorescens* as the focal species. The first microbial community used in this study consisted of the opportunistic pathogen *Serratia marcescens,* two protist enemies, the particle-feeding ciliate *Tetrahymena thermophila* and surface-feeding *Acanthamoeba castellanii*, and a parasite, the lytic bacteriophage Semad11. In order to construct a system mimicking a natural microbial community, we chose two bacteriovorous protists with different modes of feeding. Instead of assessing the individual implications of each predator on the host-parasite relationship between the bacterium and the phage, we investigated how phage-host interaction is shaped in the presence of a more complex community resembling a natural one. The second community consisted of the prey *P. fluorescens* and the predator *T. thermophila*. Two alternative hypotheses were proposed: i) if bacterial defence against phages is negatively correlated with defences against protist predators, bacteria in a multi-enemy environment are forced to allocate their limited resources between several costly defences, resulting in lowered defence against phages, and thus elevated susceptibility of bacterial populations to infection by phages (relative to phage-only environment). On the other hand, ii) if bacterial defence against phages and protists is positively correlated, the strong selective pressure posed by multiple enemies is expected to lead to the emergence of bacteria that are less vulnerable to phage infections than bacteria from the phage-only environments. The evolutionary response in bacteria was measured as susceptibility to an infection by either co-evolved or ancestral phages for bacterial populations originating from individual experimental clones. In the *Serratia* system, bacteria faced with multiple enemies were in general less susceptible to infection by phages, relative to bacteria that had evolved alone or with phages, and this was independent from whether the bacteria shared a co-evolutionary history with the phage population or not. Bacteria that had evolved with phages alone were overall more susceptible to phage infection, but less vulnerable against the phage population they had co-evolved with. Selection by both, phages alone and phages and protists together, came with a cost on the bacterial competitive ability, measured as yield of bacterial biomass in absence of phages. These results were confirmed by the *Pseudomonas* system; the bacteria that had evolved with *Tetrahymena* were less susceptible to infection by phages, compared to bacteria that had evolved alone. Our findings show that the presence of protist predators could indirectly select for lowered susceptibility to infection by lytic phages, indicating that some of the evolved anti-predatory traits can also be beneficial against phage infections.

## Methods

### Study species and selection experiment

The organisms used in this study consist of the opportunistic bacterial pathogen *Serratia marcescens* strain Db11 [25], *Pseudomonas fluorescens* strain SWB25 [26], the lytic bacteriophage Semad11 infecting *Serratia* and the phage SWB25Φ2 infecting *Pseudomonas*. As predatory protists, we used the particle-feeding ciliate *Tetrahymena thermophila* (ATCC 30008, obtained from the American Type Culture Collection) and the surface-feeding amoeba *Acanthamoeba castellanii* (strain CCAP 1501/10, obtained from the Culture Collection of Algae and Protozoa, Freshwater Biological Association, The Ferry House, Ambleside, United Kingdom).

We carried out two selection experiments where bacteria were exposed to protozoan predation. The first selection experiment with the *Serratia* systems was carried out in static batch culture microcosms (25 cm^2^ Sarstedt flasks with 15 ml of NAS [New Cereal Leaf - Page’s modified Neff’s amoebae saline] medium at 25°C. NAS medium was prepared as follows: 1 g of cereal grass powder [Aldon Corp., Avon, NY] was boiled in 1 liter of dH_2_O for 5 minutes, and then filtered through a glass fiber filter [GF/C, Whatman]. After cooling down, PAS stock solutions II and I were added, 5 ml each, and dH_2_O was used to restore a final volume of 1 liter [27-29]. Microcosms were seeded either with 1. Db11 (6.7 × 10^7^ cfu/mL), 2. Db11 (6.7 × 10^7^ cfu/mL) and Semad11 (6.7 × 10^5^ pfu/mL), or 3. Db11 (6.7 × 10^7^ cfu/mL), Semad11 (6.7 × 10^5^ pfu/mL), *T. thermophila* (6.7 × 10^2^ ind./mL) and *A. castellanii* (6.7 × 10^2^ individuals/mL). Sixteen replicate microcosms were propagated for each treatment. Microcosms were cultured for eight weeks (approximately 50 to 100 bacterial generations) [30], and resources were renewed by substituting 50% of the medium with fresh NAS each week. Four batch cultures from each treatment were destructively sampled after 1, 3, 5, and 8 weeks of co-evolution. The control treatment containing only bacteria had 18 replicate microcosms (five sampled after weeks 1 and 3, and four sampled after weeks 5 and 8). Ten bacterial clones were isolated from each microcosm at each time point for growth analyses (n = 340).

The second selection experiment was carried out in a *Pseudomonas* and *Tetrahymena* system without the phage. Details of this experiment are reported in Hiltunen and Becks [31]. In brief: the culture medium for bacteria contained M9 salts and King’s B nutrients in a 5% concentration compared to full strength medium (concentrations used: 1 g Peptone number 3 and 0.5 ml glycerol in 1 liter of dH_2_O). With this system, we conducted a 28-day long microcosm experiment, representing approximately 160 *Pseudomonas* generations. All treatments started from a single ancestral smooth colony of *Pseudomonas* (i.e. initial genetic variability in the prey population was minimized). All treatments were replicated three times in 25 ml glass vials containing 6 ml of 5% King’s B media. Every 48 hours, 2.5% of each culture was transferred into a new vial containing fresh culture medium. Microcosms were kept in 28°C (±0.1°C) with constant shaking at 50 rpm. During each transfer, both predator and prey abundances were estimated, and a 0.5 ml subsample was frozen with 0.5 ml of 80% glycerol and kept at − 80°C for later analysis. Ciliates do not survive freezing under these conditions. From the last sampling point of this experiment, we isolated 10 bacterial clones from the treatment with bacteria alone (Figure two a in Hiltunen and Becks [31]) and from the treatment with bacteria and ciliates (Figure two c in Hiltunen and Becks [31]). The rationale for testing both *Serratia* and *Pseudomonas* sp. was to confirm that results are not species-specific.

### Measuring bacterial growth ability

Bacterial growth ability was measured as optical density which is known to correlate with dry weight across different organisms [32]. In the *Serratia* system measurements were done with Bioscreen C® spectrophotometer (420–580 nm wideband filter) on 100-well “Honeycomb 2” plates (Oy Growth Curves Ab Ltd) for all isolated bacterial clones (n = 340). Approximately 10^5^ bacterial cells grown to late log phase in NAS-medium were inoculated in 400 μl of NAS medium in each well. Three replicates were used for each bacterial clone. For the *Pseudomonas* system cultures were kept at 28°C and optical density (600 nm) for all isolated clones (n = 20) was measured once after 12 hours (UV-1800 spectrophotometer, Shimadzu, Japan).

### Measuring the susceptibility of bacterial populations to infection by phages

To assess the susceptibility of bacterial populations to infection by phages, bacterial growth of isolated bacterial clones was monitored as optical density with and without phages. The susceptibility of a bacterial population (originating from a single clone) for an infection by a given phage population was measured as the difference in optical density between bacteria grown in the presence and absence of phages. For the *Serratia* system measurements were done on 100-well “Honeycomb 2” plates in 400 μl of NAS medium and measured with Bioscreen C® plate reader (420–580 nm) for 48 h at 25°C. Evolved phage populations were isolated from microcosms by centrifugation, and samples were treated with chloroform. Co-evolved phages (unknown population density) or ancestral phages (~10^5^ pfu) were inoculated simultaneously with bacterial inoculums in the measurements where phages were included.

### Data analysis

#### Bacterial growth parameters

Bacterial yield was determined as the highest arithmetic mean of untransformed OD values in the 25-point sliding time window data. OD of the background medium was subtracted from all OD values. Mean maximum yield was calculated for each clone by using the three replicates from the treatments without phage addition. Furthermore, intra-microcosm variation was controlled by calculating a mean maximum yield for each microcosm.

#### Bacterial susceptibility to phage infections, ‘Phage effect’

To quantify the susceptibility of bacteria to an infection by a phage population we created a variable “Phage effect”, describing the mean maximum decrease in optical density per time series caused by phage addition (by co-evolved or ancestral phages). For this, the optical density of bacterial growth was monitored 1) in absence or 2) presence of phages, and each clonal measurement had three replicates. The three growth curves obtained for each bacterial clone were used to create 1000 new growth curves by permutation. Thus, in these 1000 created growth curves, a single value for a given time point could originate from any of the three replicate measurements. This procedure was done to mitigate the effect of potential unexplained variation between the three replicate measurements on the ‘Phage effect’ variable. Subsequently, the 1000 new growth curves for each bacterial clone from phage-containing and phage-free treatments were superimposed, and the mean of maximum decrease in optical density caused by phage addition was designated the phage effect of a given phage type (ancestral or co-evolved). Furthermore, we calculated a mean phage effect for each of the 10 clones isolated from a given microcosm to control for intra-microcosm variation. This mean phage effect for a microcosm value was the dependent variable when comparing phage resistance between bacteria with phage-only and multi-enemy co-evolutionary histories. For the *Pseudomonas* system, we compared the difference between optical density of cultures grown alone and cultures grown with the ancestral phage.

#### Statistical analyses

The effect of time on dependent variables (ancestral and co-evolved phage effects and yield) was tested for each treatment (phage-only, multi-enemy, no enemies) using one-way ANOVA and the Games-Howell post-hoc test for multiple comparisons when needed. Bacterial phage resistance to both the co-evolved and the ancestral phage was compared between the bacteria that had co-evolved with the phage only and the bacteria that had co-evolved with multiple enemies using Mann-Whitney’s U-test. The phage resistance observed was also compared between ancestral and contemporary phage infection treatments; this was done separately for phage-only and multi-enemy bacteria, again using Mann-Whitney’s U-test. One-way ANOVA was used to see whether there were differences in yield between ancestral, phage-only, multi-enemy and no-enemies bacteria. Post-hoc multiple comparisons were performed with the Games-Howell test. All analyses were performed with IBM® SPSS® statistics, version 20.

## Results

In the *Serratia* system, the four experimental weeks did not differ in terms of co-evolved “phage effects” (phage-only: *F*_3,12_ = 0.648, *p* = 0.599; multi-enemy: *F*_3,12_ = 0.566, *p* = 0.640), ancestral phage effects (phage-only: *F*_3,12_ = 4.443, *p* = 0.026; multi-enemy: *F*_3,12_ = 1.017, *p* = 0.419), maximum growth rates (phage-only: *F*_3,12_ = 1.435, *p* = 0.281; multi-enemy: *F*_3,12_ = 0.418, *p* = 0.743; no enemies: *F*_3,14_ = 0.276, *p* = 0.842) or yields (phage-only: *F*_3,12_ = 2.997, *p* = 0.073; multi-enemy: *F*_3,12_ = 0.172, *p* = 0.913; no enemies: *F*_3,14_ = 0.104, *p* = 0.956). Therefore, the data from the four sampling points was pooled for all the response variables.

### Bacterial susceptibility to phage infections

**Figure 1:**
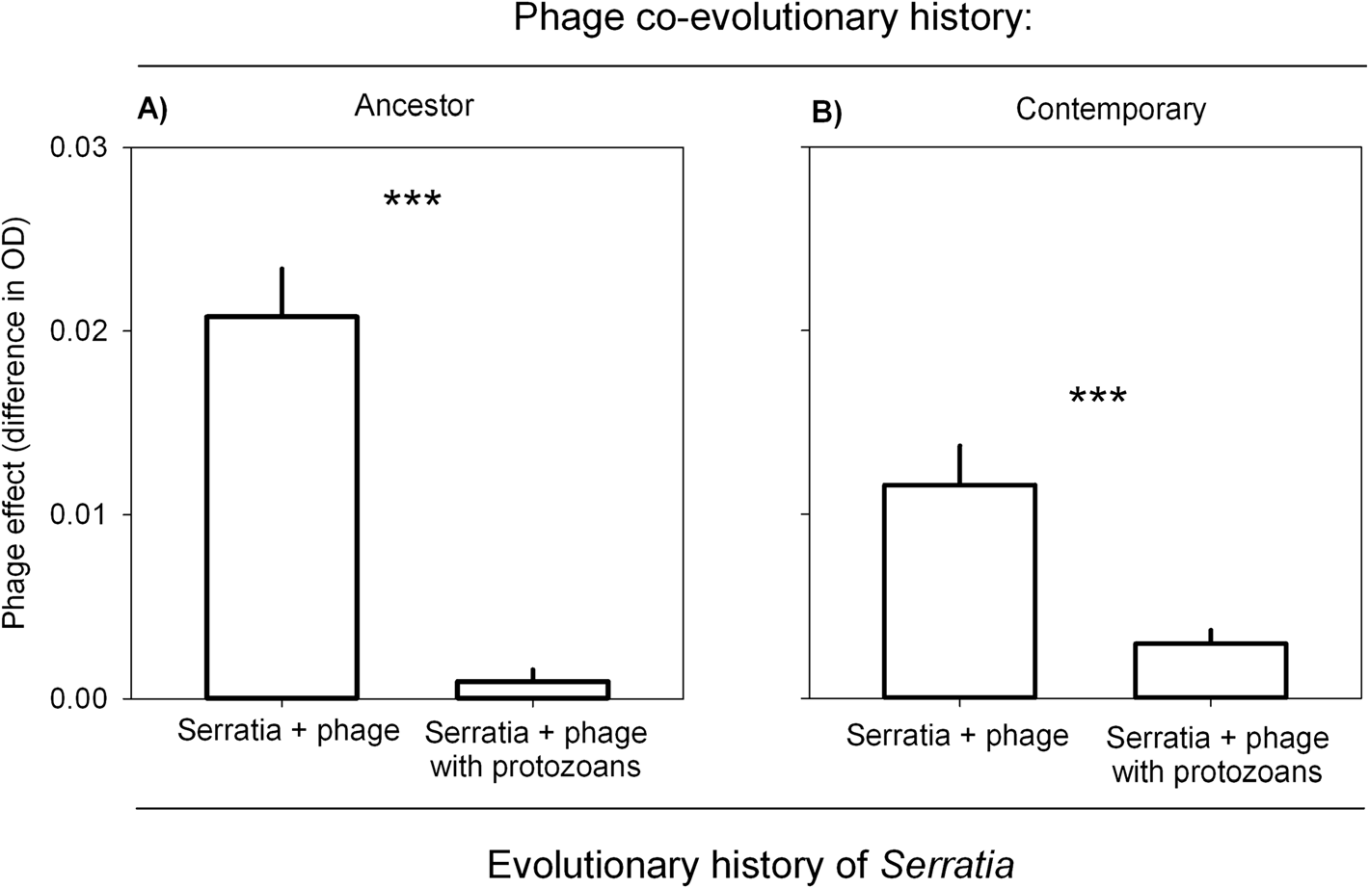
Phage effect (mean ± SE) comparisons between bacteria with different co-evolutionary histories. The smaller the phage effect value, the less susceptible bacteria are to phage infection. **A**. Infection with ancestral phage. **B**. Infection with co-evolved phage. Data from the *Serratia* system.

In the *Serratia* system, bacteria that had a co-evolutionary history with multiple enemies (phage, ciliate and amoeba) were less susceptible to infection by Semad11 than bacteria that had co-evolved with only a single phage enemy. This applied to both co-evolved (Mann-Whitney *U* = 43.0, *n* = 16, *p* = 0.001) and ancestral phages (Mann-Whitney *U* = 10.0, *n* = 16, *p* < 0.001) (Figure 1).

Bacteria with a phage-only history were less susceptible to their co-evolved phages than to the ancestral phage (Mann-Whitney *U* = 201.0, *n =* 16, *p* = 0.006). Conversely, bacteria with multi-enemy history less susceptible to the ancestral phage than to their co-evolved phages (Mann-Whitney *U* = 61.0, *n* = 16, *p* = 0.012).

**Figure 2:**
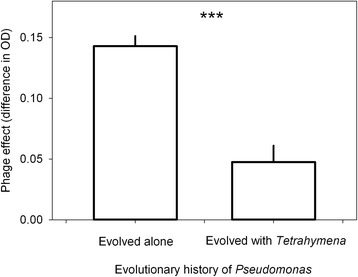
Phage effect (mean ± SE) comparisons between *Pseudomonas* with different co-evolutionary histories. The bacteria have evolved either alone or with *Tetrahymena* in a 28-day long microcosm experiment. The smaller the phage effect value, less susceptible bacteria are to phage infection.

In the *Pseudomonas* system, bacteria that had evolved with *Tetrahymena* were less susceptible to infection by phage, compared to bacteria that had evolved alone in the same conditions (Figure 2, *F*_1,4_ = 36.7; *p* = 0.004).

### Bacterial growth ability

**Figure 3:**
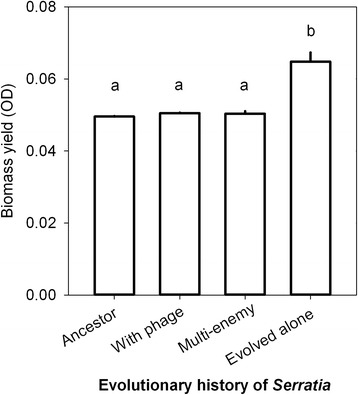
Maximum bacterial biomass (yield) comparisons between bacteria with different evolutionary histories. Letters (a–c) within two variables indicate subsets that are not different from each other. Data from the *Serratia* system.

Bacteria that had evolved in the absence of enemies had a higher yield than ancestral, phage-only or multi-enemy bacteria (*F*_3,109_ = 55.608, *p* < 0.001, Figure 3 b; ancestor vs. no enemies: *p* < 0.001; phage-only vs. no-enemies: *p* < 0.001; phage-only vs. no-enemies: *p* < 0.001). Phage-only bacteria had a higher yield compared to ancestral bacteria (*p* < 0.001).

## Discussion

In this study, we demonstrate that the presence of the particle-feeding ciliate *Tetrahymena thermophila* and surface-feeding amoeba *Acanthamoeba castellanii* selected for *Serratia marcescens* that were less vulnerable to an infection by lytic bacteriophages, in comparison to bacteria from phage-only systems (Figure 1). These results were further supported by another study system where *Pseudomonas* bacteria evolved with *Tetrahymena* ciliate (Figure 2).

*Serratia* that had evolved in the phage-only systems were more susceptible to the ancestral phage than to co-evolved phages (Figure 3). This result is in line with a study by [33] demonstrating that bacteria are most resistant to their contemporary phages in a low-productivity environment. The dominating bacterial defence against phages was thus selective for phage type, potentially through alterations in the surface structures on that the phages recognize. However, the *Serratia* that had evolved with the phages and protist predators, were less susceptible to infection by both contemporary and ancestral phages, relative to bacteria that had evolved alone. This suggests that some of the evolved anti-predatory traits could be also beneficial against phage infection, and this mechanism was less selective to phage type. One such less selective mechanism to avoid infection by phages on bacterial population level could be the production of biofilm, allowing some bacteria to hide from phages as spatial refugees [34]. Furthermore, biofilm formation has been shown to increase in bacteria in the presence of many phages [35] as well as the protist *T. thermophila* [36] and moreover, Semad11 has been shown not to have any negative long-term effects on *S. marcescens* biofilm biomass in aquatic systems [30]. In addition to biofilm formation, colony formation is known to be used as a defence against protist predation [9] and indeed, observation of liquid cultures under a light microscope indicates that both of the bacterial species used in this study evolve colony defences against grazing by *T. thermophila* within a week. In addition to phages, amoebas are also able to recognize surface structures of bacteria prior to phagocytosis [37]. One potential mechanism through which bacteria could escape both phage and amoeba predation is masking of receptors on the cell surface by producing extracellular polymer structures, providing a physical barrier between the enemies and their receptors. Low resource environments can select for bacteria with high competitive ability [38], which is likely to account for the increased bacterial yield in the *Serratia* system with no enemies. However, the evolution for increased biomass yield was constrained by the presence of phages, and phages and protists together, likely through costs associated with antipredatory and antiparasitic defence traits.

Our results can have implications beyond the eco-evolutionary community effects presented here, since many bacteria that actively grow in natural multispecies reservoirs can also opportunistically cause infections in multicellular organisms [39]. One example of this type of opportunistic bacteria is *S. marcescens* used in this study. As life in natural reservoirs and possessing virulence against multicellular hosts pose distinct challenges for bacteria, their life-history in natural reservoirs may be expected to have implications for virulence. More specifically, bacterial antipredatory defence traits are often traded off with competitive ability (indicated by growth rate or yield in bacteria) [40-44], either through pleiotropy at related genetic loci or through costs associated with resistance mechanisms [45]. Competitive ability in bacteria, in turn, is often linked to virulence, e.g. through the rate at which the host is colonized [46-48]. Phage-resistance has been shown to correlate with lowered pathogenicity in e.g. *Serratia marcescens*, *Bacillus thuringiensis, Vibrio cholerae*, *Eschericia coli*, *Klebsiella pneumoniae,* and *Salmonella* species [25,49-53]. As the presence of protists was shown to affect the outcomes of bacterial susceptibility for phage infection, along with growth ability in *S. marcescens*, our findings interestingly suggest that the enemy composition in natural reservoirs of bacterial opportunists could have implications for the virulence of opportunistically pathogenic bacteria.

## Conclusions

In conclusion, we tested how bacterial susceptibility to an infection by a parasitic phage and the potential associated cost on the competitive ability of the bacterium is modified by the presence of a community of protist bacterivores. Bacteria that had evolved with multiple enemies were overall less susceptible to infection, by both ancestral, and contemporary phages. Pairwise co-evolution with phages led to a specific defence against phages, where bacteria were more resistant to the contemporary phages than the ancestral phages. Allocation to defence was costly for the bacterium in general, constraining the evolution for increased bacterial yield in all systems. Our study is among few studies showing the implications of multiple species interactions on an evolving host-parasite system, and it suggests a previously unreported overlap of bacterial defence against predation and parasitism in microbial consumer-resource communities.

## Availability of supporting data

The data sets supporting the results of this article are available online in the Dryad data repository under doi:10.5061/dryad.s64vm [54].
